# 2-Amino-3-chloro-5-nitro­benzamide

**DOI:** 10.1107/S1600536812009142

**Published:** 2012-03-10

**Authors:** James L. Wardell, Edward R. T. Tiekink

**Affiliations:** a Centro de Desenvolvimento Tecnológico em Saúde (CDTS), Fundação Oswaldo Cruz (FIOCRUZ), Casa Amarela, Campus de Manguinhos, Av. Brasil 4365, 21040-900, Rio de Janeiro, RJ, Brazil; b Department of Chemistry, University of Malaya, 50603 Kuala Lumpur, Malaysia

## Abstract

The amide group in the title compound, C_7_H_6_ClN_3_O_3_, is significantly twisted out of the plane of the benzene ring [C—C—C—O = 34.2 (5)°] whereas the nitro group is almost co-planar [O—N—C—C = 4.0 (5)°] with the ring. Intra­molecular N—H⋯O and N—H⋯Cl hydrogen bonds occur. In the crystal, the mol­ecules are linked by N—H⋯O hydrogen bonds, generating layers propagating in the *ab* plane.

## Related literature

For crystal engineering studies on related mol­ecules, see: Wardell & Tiekink (2011 ▶).
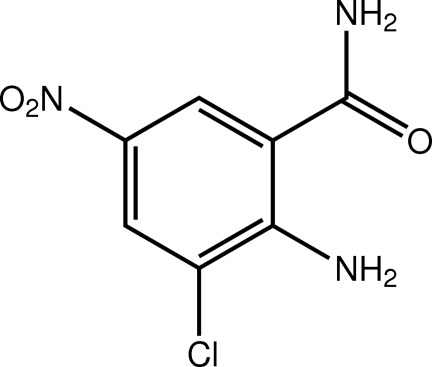


## Experimental

### 

#### Crystal data

C_7_H_6_ClN_3_O_3_
*M* *_r_* = 215.60
Triclinic, 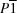

*a* = 4.891 (9) Å
*b* = 6.363 (13) Å
*c* = 14.61 (3) Å
α = 83.54 (11)°
β = 82.37 (11)°
γ = 73.64 (9)°
*V* = 431.1 (15) Å^3^
*Z* = 2
Mo *K*α radiation
μ = 0.43 mm^−1^
*T* = 100 K
0.18 × 0.08 × 0.01 mm

#### Data collection

Rigaku Saturn724+ diffractometer
Absorption correction: multi-scan (*CrystalClear-SM Expert*; Rigaku, 2011 ▶) *T*_min_ = 0.826, *T*_max_ = 1.000
3828 measured reflections
1936 independent reflections
1104 reflections with *I* > 2σ(*I*)
*R*_int_ = 0.048

#### Refinement

*R*[*F* ^2^ > 2σ(*F* ^2^)] = 0.063
*wR*(*F* ^2^) = 0.180
*S* = 0.95
1936 reflections
139 parameters
4 restraints
H atoms treated by a mixture of independent and constrained refinement
Δρ_max_ = 0.44 e Å^−3^
Δρ_min_ = −0.80 e Å^−3^

### 

Data collection: *CrystalClear-SM Expert* (Rigaku, 2011 ▶); cell refinement: *CrystalClear-SM Expert*; data reduction: *CrystalClear-SM Expert*; program(s) used to solve structure: *SHELXS97* (Sheldrick, 2008 ▶); program(s) used to refine structure: *SHELXL97* (Sheldrick, 2008 ▶); molecular graphics: *ORTEP-3* (Farrugia, 1997 ▶) and *DIAMOND* (Brandenburg, 2006 ▶); software used to prepare material for publication: *publCIF* (Westrip, 2010 ▶).

## Supplementary Material

Crystal structure: contains datablock(s) global, I. DOI: 10.1107/S1600536812009142/hb6665sup1.cif

Structure factors: contains datablock(s) I. DOI: 10.1107/S1600536812009142/hb6665Isup2.hkl

Supplementary material file. DOI: 10.1107/S1600536812009142/hb6665Isup3.cml

Additional supplementary materials:  crystallographic information; 3D view; checkCIF report

## Figures and Tables

**Table 1:** Hydrogen-bond geometry (Å, °)

*D*—H⋯*A*	*D*—H	H⋯*A*	*D*⋯*A*	*D*—H⋯*A*
N2—H3n⋯O1	0.88 (3)	2.07 (2)	2.755 (7)	134 (3)
N2—H4n⋯Cl1	0.88 (2)	2.51 (3)	2.970 (7)	113 (3)
N1—H1n⋯O1^i^	0.89 (3)	2.40 (4)	3.148 (8)	143 (3)
N1—H1n⋯O3^ii^	0.89 (3)	2.55 (3)	3.130 (8)	124 (3)
N1—H2n⋯O1^iii^	0.88 (2)	2.05 (3)	2.881 (7)	158 (4)
N2—H3n⋯O3^iv^	0.88 (3)	2.44 (4)	3.076 (8)	130 (3)
N2—H4n⋯O2^iv^	0.88 (2)	2.46 (4)	3.003 (8)	121 (3)

Symmetry codes: (i) 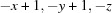; (ii) 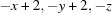; (iii) 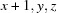; (iv) 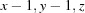.

**Fig. 1.**
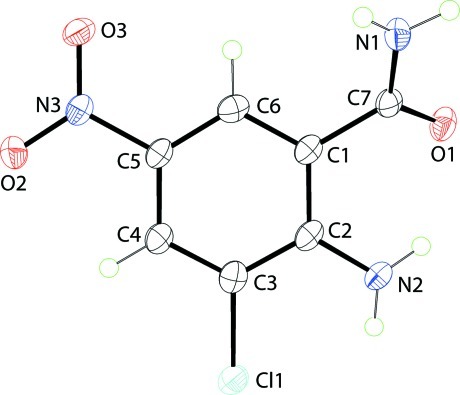
The molecular structure of (I) showing displacement ellipsoids at the 50% probability level.

**Fig. 2.**
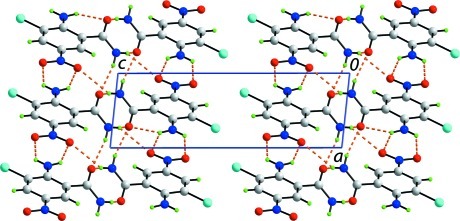
A view in projection down the b axis of the packing of supramolecular layers in (I). The N—H···O hydrogen bonds are shown as orange dashed lines.

## supplementary crystallographic information

### Comment

The crystal structure determination on the impurity found from the recrystallization of a commercially available title compound (I) was performed as a part of a programme of crystal engineering studies with small molecule acids with amine and nitro substituents (Wardell & Tiekink, 2011).

In (I), Fig. 1, the amide group is twisted out of the plane of the benzene ring to which it is connected as seen in the value of the C2—C1—C7—O1 torsion angle of 34.2 (5)°. By contrast, the nitro group is co-planar with the ring with the O3—N3—C5—C6 torsion angle being 4.0 (5)°. Both amine-H atoms form intramolecular hydrogen bonds, one to the carbonyl-O and the other the chloride substituent, Table 1. The amine-H atoms also form intermolecular interactions with each connected to a nitro-O of the same molecule for form a six-membered {···HNH···ONO}_2_ synthon. Pairs of amide groups self-associate *via* the familiar eight-membered centrosymmetric {···HNCO}_2_ synthon with this amide-H atom also connected to a translationally related amide-O atom. The second amide-H forms a hydrogen bond to a nitro-O3 atom. Thus, three of the N—H atoms form hydrogen bonds and two of the O donor atoms are bifurcated. This observation accounts for the deviations from linearity of the hydrogen bonds, Table 1. The hydrogen bonding scheme leads to the formation of layers in the *ab* plane. The layers stack along the *c* axis with no specific intermolecular interactions between them, Fig. 2.

### Experimental

The title compound was present as an impurity in a commercial batch of 2-amino-3-chloro-5-nitrobenzoic acid. It was isolated as extremely thin yellow plates from an ethanolic solution of the commercial 2-amino-3-chloro-5-nitrobenzoic acid and sodium hydroxide. IR: 3429 (*s*), 3325(*s*) and 3123(br) [NH], 1630–1586 (s, br, with maxima at 1629, 1607 and 1586) [CO], 1501(*s*) and 1317 (*s*) [NO_2_].

### Refinement

The C-bound H atoms were geometrically placed (C—H = 0.95 Å) and refined as riding with *U*_iso_(H) = 1.2*U*_eq_(C). The N-bound H-atoms were located in a difference Fourier map and refined with an N—H restraint of 0.88±0.01 Å, and with *U*_iso_(H) = 1.2*U*_eq_(N).

### Crystal data

**Table:**

C_7_H_6_ClN_3_O_3_	*Z* = 2
*M**_r_* = 215.60	*F*(000) = 220
Triclinic, *P*1	*D*_x_ = 1.661 Mg m^−^^3^
Hall symbol: -P 1	Mo *K*α radiation, λ = 0.71073 Å
*a* = 4.891 (9) Å	Cell parameters from 1104 reflections
*b* = 6.363 (13) Å	θ = 2.8–30.7°
*c* = 14.61 (3) Å	µ = 0.43 mm^−^^1^
α = 83.54 (11)°	*T* = 100 K
β = 82.37 (11)°	Plate, yellow
γ = 73.64 (9)°	0.18 × 0.08 × 0.01 mm
*V* = 431.1 (15) Å^3^	

### Data collection

**Table:**

Rigaku Saturn724+ diffractometer	1936 independent reflections
Radiation source: Rotating Anode	1104 reflections with *I* > 2σ(*I*)
Confocal monochromator	*R*_int_ = 0.048
Detector resolution: 28.5714 pixels mm^-1^	θ_max_ = 27.5°, θ_min_ = 2.8°
profile data from ω–scans	*h* = −4→6
Absorption correction: multi-scan (*CrystalClear-SM Expert*; Rigaku, 2011)	*k* = −7→8
*T*_min_ = 0.826, *T*_max_ = 1.000	*l* = −18→18
3828 measured reflections	

### Refinement

**Table:**

Refinement on *F*^2^	Primary atom site location: structure-invariant direct methods
Least-squares matrix: full	Secondary atom site location: difference Fourier map
*R*[*F*^2^ > 2σ(*F*^2^)] = 0.063	Hydrogen site location: inferred from neighbouring sites
*wR*(*F*^2^) = 0.180	H atoms treated by a mixture of independent and constrained refinement
*S* = 0.95	*w* = 1/[σ^2^(*F*_o_^2^) + (0.0912*P*)^2^] where *P* = (*F*_o_^2^ + 2*F*_c_^2^)/3
1936 reflections	(Δ/σ)_max_ < 0.001
139 parameters	Δρ_max_ = 0.44 e Å^−^^3^
4 restraints	Δρ_min_ = −0.80 e Å^−^^3^

### Special details

**Table:**

Geometry. All s.u.'s (except the s.u. in the dihedral angle between two l.s. planes) are estimated using the full covariance matrix. The cell s.u.'s are taken into account individually in the estimation of s.u.'s in distances, angles and torsion angles; correlations between s.u.'s in cell parameters are only used when they are defined by crystal symmetry. An approximate (isotropic) treatment of cell s.u.'s is used for estimating s.u.'s involving l.s. planes.
Refinement. Refinement of *F*^2^ against ALL reflections. The weighted *R*-factor *wR* and goodness of fit *S* are based on *F*^2^, conventional *R*-factors *R* are based on *F*, with *F* set to zero for negative *F*^2^. The threshold expression of *F*^2^ > 2σ(*F*^2^) is used only for calculating *R*-factors(gt) *etc*. and is not relevant to the choice of reflections for refinement. *R*-factors based on *F*^2^ are statistically about twice as large as those based on *F*, and *R*- factors based on ALL data will be even larger.

### Fractional atomic coordinates and isotropic or equivalent isotropic displacement parameters (Å^2^)

**Table:**

	*x*	*y*	*z*	*U*_iso_*/*U*_eq_	
Cl1	0.2620 (2)	0.75410 (16)	0.44557 (7)	0.0290 (3)	
O1	0.2845 (5)	0.6916 (4)	0.07122 (19)	0.0270 (7)	
O2	0.8552 (6)	1.3190 (4)	0.32947 (19)	0.0265 (7)	
O3	0.9263 (5)	1.3326 (4)	0.17747 (18)	0.0258 (7)	
N1	0.7241 (7)	0.7345 (5)	0.0176 (2)	0.0224 (7)	
H1N	0.731 (8)	0.659 (6)	−0.0305 (18)	0.027*	
H2N	0.892 (4)	0.753 (6)	0.024 (3)	0.027*	
N2	0.2414 (7)	0.6089 (5)	0.2610 (2)	0.0230 (7)	
H3N	0.201 (8)	0.579 (6)	0.2079 (15)	0.028*	
H4N	0.162 (8)	0.570 (6)	0.3155 (14)	0.028*	
N3	0.8307 (6)	1.2566 (5)	0.2533 (2)	0.0203 (7)	
C1	0.5172 (7)	0.8433 (6)	0.1718 (3)	0.0198 (8)	
C2	0.3850 (8)	0.7644 (6)	0.2572 (3)	0.0218 (8)	
C3	0.4161 (8)	0.8510 (6)	0.3398 (3)	0.0230 (9)	
C4	0.5587 (8)	1.0104 (6)	0.3399 (3)	0.0224 (8)	
H4	0.5744	1.0662	0.3961	0.027*	
C5	0.6798 (7)	1.0873 (6)	0.2546 (3)	0.0197 (8)	
C6	0.6623 (8)	1.0049 (6)	0.1714 (3)	0.0221 (8)	
H6	0.7485	1.0581	0.1148	0.026*	
C7	0.5010 (8)	0.7488 (6)	0.0829 (3)	0.0219 (8)	

### Atomic displacement parameters (Å^2^)

**Table:**

	*U*^11^	*U*^22^	*U*^33^	*U*^12^	*U*^13^	*U*^23^
Cl1	0.0357 (6)	0.0244 (6)	0.0280 (5)	−0.0122 (4)	0.0008 (4)	−0.0015 (4)
O1	0.0219 (14)	0.0255 (15)	0.0363 (16)	−0.0106 (12)	0.0018 (12)	−0.0093 (12)
O2	0.0323 (15)	0.0245 (15)	0.0253 (15)	−0.0098 (12)	−0.0041 (12)	−0.0064 (12)
O3	0.0286 (15)	0.0220 (15)	0.0270 (15)	−0.0104 (12)	0.0033 (12)	−0.0004 (12)
N1	0.0213 (16)	0.0209 (18)	0.0262 (17)	−0.0059 (14)	−0.0019 (14)	−0.0071 (14)
N2	0.0253 (17)	0.0171 (17)	0.0272 (18)	−0.0077 (14)	−0.0025 (15)	0.0006 (14)
N3	0.0173 (15)	0.0145 (16)	0.0282 (17)	−0.0025 (12)	−0.0022 (13)	−0.0027 (13)
C1	0.0157 (17)	0.0131 (18)	0.028 (2)	−0.0002 (14)	−0.0007 (15)	−0.0016 (15)
C2	0.0187 (18)	0.0106 (18)	0.032 (2)	0.0006 (14)	0.0001 (16)	−0.0020 (15)
C3	0.0200 (18)	0.0158 (19)	0.031 (2)	0.0000 (15)	−0.0067 (16)	−0.0017 (16)
C4	0.0234 (19)	0.0141 (18)	0.028 (2)	−0.0018 (15)	−0.0011 (16)	−0.0040 (16)
C5	0.0162 (17)	0.0134 (18)	0.028 (2)	−0.0022 (14)	−0.0029 (15)	−0.0008 (15)
C6	0.0216 (18)	0.0149 (19)	0.026 (2)	−0.0007 (15)	0.0015 (16)	0.0003 (15)
C7	0.0241 (19)	0.0147 (18)	0.025 (2)	−0.0037 (15)	0.0018 (16)	−0.0041 (15)

### Geometric parameters (Å, º)

**Table:**

Cl1—C3	1.753 (5)	N3—C5	1.464 (5)
O1—C7	1.250 (5)	C1—C6	1.403 (5)
O2—N3	1.254 (4)	C1—C2	1.433 (5)
O3—N3	1.244 (4)	C1—C7	1.511 (6)
N1—C7	1.340 (5)	C2—C3	1.425 (6)
N1—H1N	0.887 (10)	C3—C4	1.383 (6)
N1—H2N	0.881 (10)	C4—C5	1.406 (6)
N2—C2	1.358 (5)	C4—H4	0.9500
N2—H3N	0.881 (10)	C5—C6	1.398 (6)
N2—H4N	0.881 (10)	C6—H6	0.9500
			
C7—N1—H1N	118 (3)	C4—C3—C2	122.8 (4)
C7—N1—H2N	127 (3)	C4—C3—Cl1	118.7 (3)
H1N—N1—H2N	112 (4)	C2—C3—Cl1	118.4 (3)
C2—N2—H3N	117 (3)	C3—C4—C5	118.1 (4)
C2—N2—H4N	118 (3)	C3—C4—H4	121.0
H3N—N2—H4N	124 (4)	C5—C4—H4	121.0
O3—N3—O2	123.2 (3)	C6—C5—C4	121.7 (4)
O3—N3—C5	119.0 (3)	C6—C5—N3	119.4 (3)
O2—N3—C5	117.8 (3)	C4—C5—N3	118.9 (3)
C6—C1—C2	120.0 (4)	C5—C6—C1	119.9 (4)
C6—C1—C7	120.7 (3)	C5—C6—H6	120.0
C2—C1—C7	119.3 (3)	C1—C6—H6	120.0
N2—C2—C3	120.4 (4)	O1—C7—N1	122.2 (4)
N2—C2—C1	122.2 (4)	O1—C7—C1	120.6 (3)
C3—C2—C1	117.4 (4)	N1—C7—C1	117.2 (3)
			
C6—C1—C2—N2	179.8 (3)	O3—N3—C5—C6	4.0 (5)
C7—C1—C2—N2	−1.0 (5)	O2—N3—C5—C6	−176.4 (3)
C6—C1—C2—C3	−2.0 (5)	O3—N3—C5—C4	−176.6 (3)
C7—C1—C2—C3	177.3 (3)	O2—N3—C5—C4	2.9 (5)
N2—C2—C3—C4	−179.6 (3)	C4—C5—C6—C1	1.0 (5)
C1—C2—C3—C4	2.1 (5)	N3—C5—C6—C1	−179.7 (3)
N2—C2—C3—Cl1	−0.6 (5)	C2—C1—C6—C5	0.5 (5)
C1—C2—C3—Cl1	−178.9 (3)	C7—C1—C6—C5	−178.7 (3)
C2—C3—C4—C5	−0.7 (6)	C6—C1—C7—O1	−146.6 (4)
Cl1—C3—C4—C5	−179.7 (3)	C2—C1—C7—O1	34.2 (5)
C3—C4—C5—C6	−0.9 (5)	C6—C1—C7—N1	31.9 (5)
C3—C4—C5—N3	179.8 (3)	C2—C1—C7—N1	−147.3 (3)

### Hydrogen-bond geometry (Å, º)

**Table:**

*D*—H···*A*	*D*—H	H···*A*	*D*···*A*	*D*—H···*A*
N2—H3n···O1	0.88 (3)	2.07 (2)	2.755 (7)	134 (3)
N2—H4n···Cl1	0.88 (2)	2.51 (3)	2.970 (7)	113 (3)
N1—H1n···O1^i^	0.89 (3)	2.40 (4)	3.148 (8)	143 (3)
N1—H1n···O3^ii^	0.89 (3)	2.55 (3)	3.130 (8)	124 (3)
N1—H2n···O1^iii^	0.88 (2)	2.05 (3)	2.881 (7)	158 (4)
N2—H3n···O3^iv^	0.88 (3)	2.44 (4)	3.076 (8)	130 (3)
N2—H4n···O2^iv^	0.88 (2)	2.46 (4)	3.003 (8)	121 (3)

Symmetry codes: (i) −*x*+1, −*y*+1, −*z*; (ii) −*x*+2, −*y*+2, −*z*; (iii) *x*+1, *y*, *z*; (iv) *x*−1, *y*−1, *z*.
